# Progress toward the UNAIDS 90–90-90 targets among female sex workers and sexually exploited female adolescents in Juba and Nimule, South Sudan

**DOI:** 10.1186/s12889-022-12533-1

**Published:** 2022-01-19

**Authors:** Avi J. Hakim, Alex Bolo, Kelsey C. Coy, Victoria Achut, Joel Katoro, Golda Caesar, Richard Lako, Acaga Ismail Taban, Katrina Sleeman, Jennifer Wesson, Alfred G. Okiria

**Affiliations:** 1 US Centers for Disease Control and Prevention, Division of Global HIV and TB, 1600 Clifton Rd, NE, US1-2, Atlanta, GA 30329 USA; 2 South Sudan Ministry of Health, Juba, South Sudan; 3 IntraHealth International, Chapel Hill, NC USA

**Keywords:** Female sex workers, Sexually exploited minors, HIV, South Sudan, 90–90-90 targets, Respondent-driven sampling

## Abstract

**Background:**

Little is known about HIV in South Sudan and even less about HIV among female sex workers (FSW). We characterized progress towards UNAIDS 90–90-90 targets among female sex workers (FSW) and sexually exploited female adolescents in Juba and Nimule, South Sudan.

**Methods:**

We conducted a biobehavioral survey of FSW and sexually exploited female adolescents using respondent-driven sampling (RDS) in Juba (November 2015–March 2016) and in Nimule (January–March 2017) to estimate achievements toward the UNAIDS 90–90-90 targets (90% of HIV-positive individuals know their status; of these, 90% are receiving antiretroviral therapy [ART]; and of these, 90% are virally suppressed). Eligibility criteria were girls and women who were aged ≥15 years; spoke English, Juba Arabic, or Kiswahili; received money, goods, or services in exchange for sex in the past 6 months; and resided, worked, or socialized in the survey city for ≥1 month. Data were weighted for RDS methods.

**Results:**

We sampled 838 FSW and sexually exploited female adolescents in Juba (HIV-positive, 333) and 409 in Nimule (HIV-positive, 108). Among HIV-positive FSW and sexually exploited female adolescents living in Juba, 74.8% self-reported being aware of their HIV status; of these, 73.3% self-reported being on ART; and of these, 62.2% were virally suppressed. In Nimule, 79.5% of FSW and sexually exploited female adolescents living with HIV self-reported being aware of their HIV status; of these, 62.9% self-reported being on ART; and of these, 75.7% were virally suppressed.

**Conclusions:**

Although awareness of HIV status is the lowest of the 90–90-90 indicators in many countries, treatment uptake and viral suppression were lowest among FSW and sexually exploited female adolescents in South Sudan. Differentiated service delivery facilitate linkage to and retention on treatment in support of attainment of viral suppression.

## Background

Key populations have a higher risk of HIV acquisition and higher HIV prevalence than the general population and simultaneously have lower access to HIV services [1–4]. Key populations and their partners account for over half of new HIV infections globally despite accounting for only a fraction of the population [5]. For female sex workers (FSW) in particular, HIV prevalence has been associated with laws banning sex work [6]. Other factors that increase risk and vulnerability of sex workers and hinder utilization of HIV services include STIs, depression, and alcohol consumption. Sex work has further been found to contribute to epidemic dynamics even in high HIV prevalence settings, reinforcing the notion that HIV services need to reach all populations [7].

The Joint United National Programme on HIV/AIDS (UNAIDS) call for diagnosing 90% of all people living with HIV (PLHIV), sustaining 90% of them on antiretroviral therapy (ART), and maintaining viral suppression among 90% of those receiving ART [8]. Although UNAIDS targets are set for all populations, global efforts to reach and measure progress toward these targets have focused on the general population [9–11]. Consequently, little is known about progress toward reaching 90–90-90 targets among key populations, including FSW. Because stigma hinders HIV service utilization and may prompt key population members who access them to conceal their behaviors, program data alone are insufficient to characterize the epidemic among key populations [12].

HIV prevalence among individuals aged 15–49 years in South Sudan is estimated at 2.4%, and there are approximately 194,000 PLHIV, less than one in five of whom are on ART [13]. HIV prevalence among FSW in Juba, the capital, was estimated at 37.9% and at 24.0% in Nimule, on the major border crossing with Uganda [14, 15]. Syphilis prevalence was 7.3% in Juba and 9.2% in Nimule [14, 15]. The illegal nature of sex work in South Sudan exposes FSW to arrest and harassment from police and other security forces, in addition to violence from clients [16, 17]. Approximately two-thirds of FSW in Juba and one-quarter in Nimule were foreign, adding further stigma and barriers to service utilization [14, 15].

Though a country for less than 10 years, South Sudan has moved quickly to develop its HIV surveillance system to inform the government’s HIV response. We present findings from the Eagle Survey of FSW and sexually exploited female adolescents in Juba and Nimule and characterize progress toward reaching the UNAIDS 90–90-90 targets among this population.

## Methods

### Study population, setting, and design

The Eagle Survey used respondent-driven sampling (RDS) to recruit FSW and sexually exploited female adolescents in Juba (November 2015–March 2016) and in Nimule (January–March 2017). RDS was chosen to recruit participants because sex work is illegal and sex workers stigmatized, FSW had social connections across sub-groups, and for the safety of FSW and study staff. Sexually exploited female adolescents were defined as those aged 15–17 years who were engaged in sex in exchange for goods, money, or services. RDS is a variant of snowball sampling used to produce sampling weights and approximate a random sample [18–21]. Eligibility criteria were girls and women who were aged ≥15 years; spoke English, Juba Arabic, or Kiswahili; received money, goods, or services in exchange for sex in the past 6 months; and resided, worked, or socialized in the survey city for at least the last 1 month.

### Recruitment and data collection

Data collection began with four seeds selected based on age, neighborhood, nationality, and influence among peers. Five more seeds were later added to reach underrepresented populations. After completing eligibility screening and providing verbal informed consent, participants underwent a face-to-face computer-assisted personal interview (Open Data Kit, Washington, US). Interview domains included demographics, experience of stigma, HIV knowledge, sexual history, uptake of HIV and sexually health services, and history of sexually transmitted diseases. The two-item Patient Health Questionnaire (PHQ-2) was used to screen for depression, and the three-question Alcohol Use Disorders Identification Test was used to screen for alcohol disorders [22, 23]. Determination of comprehensive knowledge of HIV utilized the UNAIDS definition [24].

Upon completion of the interview, consenting participants were tested for HIV by trained staff according to the national testing algorithm of Determine HIV-1/2 (Alere, MA, USA) followed by Uni-Gold (Trinity Biotech, Ireland) for reactive results to confirm HIV infection. Bioline (Standard Diagnostics, South Korea) was used as a tiebreaker for discrepant results. Participants with HIV received CD4 enumeration with the PIMA analyzer (Alere, MA, USA). Quality control of HIV-positive specimens was performed with Geenius HIV-1/2 (Bio-Rad, CA) at the South Sudan National Public Health Lab. Viral load testing was performed on dried blood spot samples at US Centers for Disease Control and Prevention (CDC; Atlanta, GA) using the Abbott RealTime HIV-1 assay (Abbott Molecular, IL) on the Abbott m2000 fully-automated platform.

Syphilis testing was conducted using Bioline syphilis 3.0 followed by the Rapid Plasma Reagin (RPR) test. Participants testing positive for HIV were given a referral letter to ART sites for treatment; those testing positive for syphilis were treated at the study site. Staff were trained to refer all girls aged younger than 18 years to partner organizations providing psychosocial and other protection services for sexually exploited minors.

Participants were compensated 60 South Sudanese Pounds (SSP, approximately $20 USD at the time of data collection) and given three coupons to recruit peers. At the second study visit they received 20 SSP for transportation and 15 SSP for each successful recruit (approximately $21 USD maximum).

### Data analysis

The primary endpoint for our analysis was awareness of HIV-positive status. The analysis was restricted to HIV-positive participants, and suppressed HIV viral load was defined as < 1000 copies/mL. Estimates of geometric mean viral load excluded values below the limit of detection (831 copies/mL) and those with a result of target not detected to avoid underestimating the mean. Estimates of median viral load do include values below the limit of detection (831 copies/mL) and those with a result of target not detected (TND).

Data were analyzed using RDS Analyst version 0.62 (Los Angeles, CA) using Gile’s Successive Sampling Estimator and SAS v9.4. Network size was determined through a series of questions that ultimately asked participants about the number of other female sex workers aged ≥15 years they knew in the study city and had seen in the last month. Diagnostics were conducted to assess the sample’s independence from seeds. RDS sampling weights were used in the calculation of odds ratios (OR) and 95% confidence intervals (CI) for bivariate comparisons, and variables significant at *p* < 0.1 were included in the weighted multivariable model.

We explored the impact of self-reporting on cascade estimates and present the 90–90-90 conditional cascade in two ways: 1) where inclusion in a step is based on self-reported response to the previous step and 2) where all participants with suppressed HIV viral load are considered to also be aware of their status and on antiretroviral therapy, with conditional cascade measures modified accordingly.

### Ethical approval

Ethical approval for the Eagle Survey was obtained from the South Sudan Ministry of Health Ethical Review Board. The protocol was also reviewed by the CDC Center for Global Health Associate Director for Science in accordance with CDC human research protection procedures and was determined to be research, but CDC investigators did not interact with human subjects or have access to identifiable data or specimens for research purposes. The South Sudan Ministry of Health Ethical Review Board and the CDC Center for Global Health Associate Director for Science approved of the use of verbal informed consent because written consent would be the only identifiable information collected and could pose a risk to participants. A waiver to obtain informed consent from parents or guardians of participants under the age of 18 was granted as the risks of participation were minimal and outweighed by the potential risks of disclosure of sex work to parents or guardians. All methods were performed in accordance with the relevant guidelines and regulations.

## Results

**Fig. 1:**
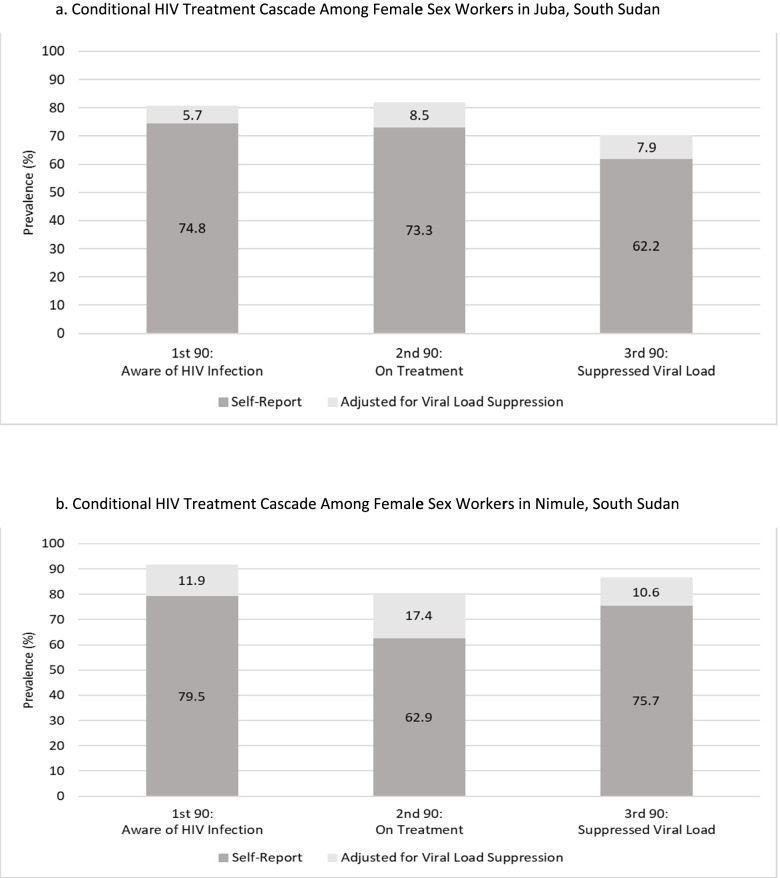
**a** Conditional HIV Treatment Cascade Among Female Sex Workers in Juba, South Sudan. **b**. Conditional HIV Treatment Cascade Among Female Sex Workers in Nimule, South Sudan

We sampled 838 FSW and sexually exploited female adolescents in Juba (HIV-positive, 333) and 409 in Nimule (HIV-positive, 108). Sexually exploited female adolescents were defined as those aged 15–17 years who were engaged in sex in exchange for goods, money, or services. Among FSW and sexually exploited female adolescents living with HIV in Juba, 74.8% self-reported being aware of their HIV status; of these, 73.3% self-reported being on ART; of these, 62.2% were virally suppressed (Fig. 1a). When adjusted for viral suppression, 80.5% of FSW and sexually exploited female adolescents were aware of their HIV status; of these, 81.8% were on ART; and of these, 70.1% were virally suppressed. Viral suppression among all FSW and sexually exploited female adolescents living with HIV in Juba, regardless of awareness and ART status, was 41.1% (95% CI: 34.4–47.8). The geometric mean viral load among those with measurable viral load was 8844 copies/mL (95% CI: 6589-11,871), and 59.3% (95% CI: 52.5–66.0) of all FSW and sexually exploited female adolescents in Juba had VL ≥1000 copies/mL. Median viral load was 2318 copies/mL (interquartile range [IQR], 831–22,908).

In Nimule, 79.5% of FSW and sexually exploited female adolescents living with HIV self-reported being aware of their HIV status; of these, 62.9% self-reported being on ART; of these, 75.7% were virally suppressed (Fig. 1b). When adjusted for viral suppression, 91.4% of FSW and sexually exploited female adolescents were aware of their HIV status; of these, 80.3% were on ART; and of these, 86.3% were virally suppressed. Viral suppression among all FSW and sexually exploited female adolescents living with HIV in Nimule, regardless of awareness and ART status, was 52.8% (95% CI: 41.7–63.9). The geometric mean viral load among those with measurable viral load was 9955 copies/mL (95% CI: 5726-17,309), and 47.2% (95% CI: 36.1–58.4) of all FSW and sexually exploited female adolescents in Nimule had VL ≥1000 copies/mL. Median viral load was 831 copies/mL (IQR, TND-22,908).

Among FSW and sexually exploited female adolescents living with HIV in Juba, awareness of HIV status was 81.0% among those aged 15–24 years and 72.6% among those aged ≥35 years (Table 1). In Nimule, 90.7% of those aged 15–24 years and 95.6% of those aged ≥35 years were aware of their HIV status. Although 62.3% of South Sudanese FSW and sexually exploited female adolescents in Juba were aware of their HIV status, in Nimule 100.0% were. Non-Sudanese accounted for 85.7% of HIV-positive FSW and sexually exploited female adolescents in Juba and 70% in Nimule. Awareness among non-South Sudanese FSW and sexually exploited female adolescents was similar in both cities (Juba, 82.4%; Nimule, 88.6%). In Juba, awareness was higher (90.9%) among those who had been away from home for ≥1 month in the last 6 months compared to those who had not (76.5%). In Nimule, awareness was 83.6% for those who have been away for ≥1 month and was 95.3% for those who had not. In Juba, nearly two-thirds of those who were ashamed of selling sex (65.6%) were aware of their HIV status compared to 84.2% of those who were not ashamed. In contrast, in Nimule, 94.5% of those who were ashamed of selling sex were aware of their status compared to 86.5% of those who were not ashamed.

**Table 1:** Characteristics of HIV-positive female sex workers at each stage of the conditional 90–90-90 cascade in Juba and Nimule, South Sudan, 2015–2016

	Juba				Nimule			
	HIV Positive	HIV-positive aware ^a^	On treatment ^a^	Suppressed viral load	HIV Positive	HIV-positive aware ^a^	On treatment^a^	Suppressed viral load
	***N*** = 333	***N*** = 233	***N*** = 190	***N*** = 126	***N*** = 108	***N*** = 82	***N*** = 69	***N*** = 58
	n (%; 95% CI)	n (%; 95% CI)	n (%; 95% CI)	n (%; 95% CI)	n (%; 95% CI)	n (%; 95% CI)	n (%; 95% CI)	n (%; 95% CI)
Age (years)								
15–24	33 (11.1; 6.7–15.5)	22 (81.0; 65.6–96.5)	14 (59.6; 35.0–84.2)	10 (80.3; 55.9–100.0)	15 (14.6; 6.5–22.6)	10 (90.7; 74.8–100.0)	5 (51.1; 15.2–86.9)	4 (100.0; 88.4–100.0)
25–34	192 (58.6; 52.0–65.3)	139 (84.7; 78.5–90.9)	114 (83.1; 75.9–90.3)	70 (65.6; 55.6–75.7)	60 (55.5; 44.6–66.4)	46 (89.6; 80.7–98.5)	39 (81.8; 67.7–96.0)	31 (80.0; 66.7–94.1)
≥ 35	102 (30.3; 24.0–38.6)	70 (72.6; 61.4–83.8)	61 (89.4; 81.5–97.4)	45 (75.3; 62.3–88.3)	33 (29.9; 19.9–40.0)	26 (95.6; 86.9–100.0)	25 (92.5; 78.2–100.0)	23 (94.9; 84.9–100.0)
Education								
No formal education	133 (45.1; 38.5–51.7)	88 (79.1; 70.7–87.4)	71 (82.0; 72.9–91.1)	48 (71.4; 59.7–83.1)	57 (57.1; 46.4–67.7)	41 (98.7; 96.1–100.0)	36 (85.7; 71.9–99.4)	32 (86.9; 74.3–99.5)
Primary	123 (35.2; 28.8–41.5)	89 (81.4; 72.7–90.2)	71 (79.4; 68.8–90.1)	45 (69.7; 56.9–82.5)	34 (28.5; 19.0–38.1)	28 (89.8; 79.5–100.0)	23 (76.8; 56.3–97.4)	17 (81.3; 63.8–98.8)
High school or higher	73 (19.7; 14.7–24.7)	55 (81.8; 70.9–92.7)	47 (85.5; 74.6–96.3)	32 (67.6; 51.0–84.2)	17 (14.4; 6.9–21.9)	13 (72.8; 47.1–98.6)	10 (67.1; 34.6–99.5)	9 (100.0)
Ever married								
Yes	265 (82.7; 78.0–87.5)	188 (81.0; 75.1–86.9)	153 (83.2; 77.2–89.3)	100 (70.6; 62.2–79.0)	91 (83.3; 75.0–91.7)	70 (90.5; 83.3–97.6)	62 (86.9; 76.5–97.2)	51 (84.6; 74.1–95.0)
No	64 (17.3; 12.5–22.0)	44 (78.1; 65.8–90.4)	36 (75.1; 57.5–92.6)	25 (66.9; 48.3–85.5)	17 (16.7; 8.3–25.0)	12 (95.8; 87.3–100.0)	7 (49.4; 17.1–81.8)	7 (100.0; 92.4–100.0)
Nationality								
South Sudanese	42 (14.3; 9.7–18.9)	15 (62.3; 40.1–84.4)	12 (72.5; 45.4–99.5)	9 (67.1; 34.3–99.8)	28 (30.0; 19.6–40.3)	18 (100.0; 97.4–100.0)	16 (80.1; 55.5–100.0)	14 (93.8; 81.6–100.0)
Other	287 (85.7; 81.1–90.3)	217 (82.4; 77.1–87.7)	177 (82.5; 76.5–88.5)	116 (70.2; 62.3–78.1)	80 (70.0; 59.7–80.4)	64 (88.6; 80.5–96.6)	53 (80.3; 68.2–92.5)	44 (83.5; 71.6–95.4)
Away from home for ≥1 month, past 6 months								
Yes	80 (25.4; 19.2–31.6)	67 (90.9; 84.5–97.2)	58 (85.7; 74.6–96.9)	39 (74.8; 61.4–88.2)	37 (32.1; 22.3–41.9)	26 (83.6; 68.9–98.3)	21 (85.6; 72.1–99.2)	17 (82.3; 64.8–99.9)
No	249 (74.6; 68.4–80.8)	165 (76.5; 69.8–83.2)	131 (80.0; 73.0–87.0)	86 (67.7; 58.5–76.9)	71 (67.1; 58.1–77.7)	56 (95.3; 90.1–100.0)	48 (77.9; 63.4–92.5)	41 (88.3; 77.4–99.2)
Screened positive for depression								
Yes	134 (39.5; 33.0–46.1)	92 (78.7; 69.6–87.7)	80 (87.8; 80.0–95.5)	56 (74.3; 63.0–85.5)	41 (40.4; 29.6–51.2)	32 (94.8; 85.5–100.0)	30 (89.8; 75.0–100.0)	28 (94.1; 82.5–100.0)
No	193 (60.5; 53.9–67.0)	139 (82.2; 75.8–88.7)	108 (78.0; 69.8–86.3)	68 (66.9; 56.7–77.2)	66 (59.6; 48.8–70.4)	49 (89.0; 80.7–97.2)	38 (73.2; 57.8–88.6)	29 (79.6; 65.4–93.8)
Screened positive for alcohol abuse, AUDIT-C								
Yes	122 (36.6; 30.4–42.9)	94 (85.4; 77.8–92.9)	76 (80.2; 70.0–90.3)	48 (64.7; 52.2–77.2)	46 (39.8; 29.3–50.5)	34 (88.6; 76.8–100.0)	25 (70.9; 51.8–89.9)	22 (88.0; 70.9–100.0)
No	206 (63.4; 57.1–69.6)	137 (77.6; 70.4–84.7)	112 (82.8; 75.5–90.1)	76 (73.3; 63.7–82.9)	62 (60.1; 49.5–70.7)	48 (93.2; 86.6–99.7)	44 (86.1; 72.9–99.4)	36 (85.5; 74.3–96.7)
Tested positive for syphilis								
Yes	53 (14.9; 10.4–19.3)	38 (87.9; 78.4–97.5)	34 (87.5; 75.4–99.5)	29 (82.3; 66.3–98.3)	28 (26.3; 16.8–35.7)	24 (99.1; 97.2–100.0)	19 (83.4; 68.7–98.1)	15 (78.2; 55.8–100.0)
No	280 (85.1; 80.7–89.6)	195 (79.4; 73.5–85.3)	156 (80.9; 74.3–87.5)	97 (67.8; 59.2–76.4)	79 (73.7; 64.3–83.2)	58 (88.6; 80.4–96.9)	50 (79.1; 64.9–93.2)	43 (89.8; 80.9–98.6)
STI symptoms, last 12 months								
Yes	162 (47.1; 40.4–53.7)	124 (84.0; 76.9–91.0)	102 (82.0; 73.7–90.3)	68 (72.0; 61.8–82.3)	18 (17.4; 9.3–25.5)	14 (82.9; 61.8–100.0)	11 (78.5; 55.2–100.0)	11 (100.0; 93.9–100.0)
No	162 (52.9; 46.3–59.6)	104 (76.7; 68.7–84.7)	84 (82.4; 74.0–90.8)	55 (68.0; 56.4–79.6)	85 (82.6; 74.5–90.7)	64 (93.4; 87.6–99.2)	55 (79.8; 66.9–92.8)	44 (82.6; 70.8–94.3)
Disclosed sex work to anyone ^b^								
Yes	81 (23.5; 18.1–28.8)	53 (71.0; 58.9–83.1)	41 (78.2; 65.7–90.7)	27 (67.8; 51.4–84.2)	21 (24.8; 14.6–34.9)	18 (97.5; 92.4–100.0)	15 (71.7; 45.3–98.1)	13 (93.8; 81.7–100.0)
No	247 (76.5; 71.2–81.9)	178 (83.5; 77.8–89.2)	147 (82.7; 76.0–89.5)	97 (70.5; 61.8–79.2)	87 (75.2; 65.1–85.4)	64 (89.0; 80.7–97.2)	54 (84.0; 73.7–94.3)	45 (83.5; 71.6–95.4)
Ashamed of selling sex								
Yes	73 (21.5; 16.2–26.8)	42 (65.6; 50.8–80.5)	33 (77.3; 60.1–94.5)	20 (60.9; 40.6–81.1)	65 (59.3; 48.5–70.2)	50 (94.5; 88.5–100.0)	42 (79.7; 65.7–93.8)	37 (87.0; 74.6–99.4)
No	254 (78.5; 73.2–83.8)	189 (84.2; 78.9–89.5)	155 (82.6; 76.3–88.9)	104 (71.6; 63.3–79.8)	42 (40.7; 29.8–51.5)	31 (86.5; 74.1–98.9)	26 (81.0; 62.8–99.1)	21 (86.4; 72.3–100.0)
Ashamed to disclose sex work to health worker								
Yes	127 (38.4; 32.0–44.7)	83 (76.6; 67.8–85.5)	68 (80.8; 70.4–91.3)	43 (62.3; 48.4–76.2)	71 (67.0; 56.4–77.5)	54 (94.0; 88.2–99.9)	45 (79.0; 65.5–92.4)	39 (85.5; 73.3–97.7)
No	199 (61.6; 55.3–68.0)	147 (82.9; 76.2–89.5)	119 (82.2; 75.0–89.5)	81 (74.3; 65.5–83.2)	38 (33.0; 22.5–43.6)	27 (86.1; 72.1–100.0)	23 (82.8; 63.2–100.0)	19 (89.6; 76.6–100.0)
Distance traveled to closest ART facility (km)								
0 - < 1.5	77 (53.9; 43.4–64.5)	60 (89.4; 81.3–97.5)	50 (86.0; 76.1–95.9)	31 (67.6; 52.5–82.7)				
≥ 1.5	65 (46.1; 35.5–56.6)	49 (83.9; 73.5–94.3)	39 (75.7; 60.7–90.6)	27 (80.0; 65.7–94.4)				
Median (IQR)	1.4 (0.7–2.3)	1.4 (0.7–2.4)	1.3 (0.7–2.0)	1.4 (0.7–2.2)	8.8 (6.1–9.0)	8.8 (6.0–9.2)	8.7 (5.8–9.0)	8.0 (5.7–9.0)

Abbreviations: *CI* confidence interval; *AUDIT-C* Alcohol Use Disorders Identification Test; *STI* sexually transmitted infection; *ART* antiretroviral therapy; *IQR* interquartile range

^a^ Self-reported

^b^ Includes immediate and extended family members, spouse or partner, friends who do not sell sex, health care providers, and other

Among FSW and sexually exploited female adolescents living with HIV, 44.6% in Juba and 39.6% in Nimule had sold sex for ≤2 years (Table 2). Among those who used a condom at last sex, awareness of HIV status was 82.4% in Juba and 88.7% in Nimule. Among those who did not use a condom, awareness of HIV status was 61.9% in Juba and 98.9% in Nimule. Among those who spoke with a peer educator or outreach worker in the last 12 months, awareness of HIV status was 86.4% in Juba and 93.3% in Nimule. Awareness of HIV status was higher among those in Juba who received free condoms in the last 12 months (80.8%; Nimule, 87.4%) than among those who had not (59.7%; Nimule, 96.6%).

**Table 2:** Sex work characteristics, sexual behaviors, and HIV services among HIV-positive female sex workers at each stage of the conditional 90–90-90 cascade in Juba and Nimule, South Sudan, 2016

	Juba				Nimule			
	HIV Positive	HIV-positive aware ^**a**^	On treatment ^**a**^	Suppressed viral load	HIV Positive	HIV-positive aware ^**a**^	On treatment ^**a**^	Suppressed viral load
	***N*** = 333	***N*** = 233	***N*** = 190	***N*** = 126	***N*** = 108	***N*** = 82	***N*** = 69	***N*** = 58
	n (%; 95% CI)	n (%; 95% CI)	n (%; 95% CI)	n (%; 95% CI)	n (%; 95% CI)	n (%; 95% CI)	n (%; 95% CI)	n (%; 95% CI)
Years selling sex								
≤ 2 years	138 (44.6; 37.9–51.3)	106 (83.6; 75.8–91.4)	88 (83.3; 75.0–91.6)	68 (80.7; 71.6–89.8)	40 (39.6; 28.8–50.5)	28 (83.6; 70.1–97.1)	22 (71.0; 49.2–92.9)	18 (86.0; 69.3–100.0)
3–4 years	88 (25.5; 19.8–31.1)	67 (83.6; 74.8–92.4)	49 (72.4; 59.0–85.8)	26 (57.0; 40.6–73.4)	32 (27.7; 18.3–37.1)	25 (93.0; 84.3–100.0)	22 (91.9; 81.6–100.0)	19 (90.6; 78.9–100.0)
≥ 5 years	96 (29.9; 24.0–35.9)	55 (72.9; 61.6–84.2)	48 (88.9; 79.8–97.9)	29 (62.1; 45.4–78.7)	36 (32.7; 22.6–42.8)	29 (100.0)	25 (80.4; 62.1–98.6)	21 (82.2; 63.3–100.0)
Has someone who facilitates meeting clients								
Yes	75 (23.0; 17.5–28.4)	49 (84.3; 73.3–95.4)	39 (79.8; 66.8–92.8)	26 (72.0; 55.1–88.8)	36 (28.5; 19.2–37.7)	30 (89.3; 78.6–100.0)	24 (77.2; 59.6–94.8)	19 (83.1; 66.0–100.0)
No	252 (77.0; 71.6–82.5)	182 (79.5; 73.4–85.5)	149 (82.3; 75.6–89.0)	98 (69.4; 60.7–78.1)	72 (71.5; 62.3–80.8)	52 (92.4; 84.9–99.9)	45 (81.7; 67.7–95.8)	39 (87.8; 76.6–99.0)
Number of male clients who gave money, last 6 months								
< 5	13 (6.1; 2.3–9.9)	9 (86.3; 60.9–100.0)	7 (94.7; 86.4–100.0)	2 (48.2; 4.3–92.0)	12 (18.5; 7.5–29.5)	6 (100.0)	5 (91.8; 74.7–100.0)	5 (100.0; 90.5–100.0)
≥ 5	188 (93.9; 90.1–97.7)	125 (76.7; 69.1–84.2)	104 (84.5; 77.4–91.6)	71 (71.2; 61.2–81.2)	45 (81.5; 70.5–92.5)	37 (97.8; 94.4–100.0)	32 (81.8; 62.3–98.3)	29 (90.2; 78.4–100.0)
Used condoms with all male clients, last 6 months								
Yes	192 (61.2; 54.7–67.6)	152 (85.0; 79.1–91.0)	126 (83.5; 76.8–90.2)	80 (69.5; 60.0–79.0)	24 (20.6; 12.0–29.3)	18 (89.0; 75.2–100.0)	13 (69.8; 45.2–94.4)	8 (58.0; 23.1–92.9)
No	128 (38.8; 32.4–45.3)	74 (72.2; 62.0–82.5)	57 (76.7; 64.4–89.0)	43 (74.0; 60.6–87.3)	78 (79.4; 70.7–88.0)	62 (91.6; 84.4–98.8)	54 (82.0; 69.0–94.9)	48 (90.5; 81.9–99.2)
Used condom at last sex act								
Yes	290 (87.9; 83.7–92.2)	213 (82.4; 77.0–87.6)	177 (83.7; 77.8–89.6)	117 (69.5; 61.5–77.5)	78 (70.5; 60.1–80.9)	61 (88.7; 80.6–96.9)	50 (80.0; 67.6–92.3)	41 (83.1; 71.0–95.3)
No	38 (12.1; 7.8–16.3)	18 (61.9; 39.7–84.0)	11 (56.2; 28.4–84.1)	7 (77.5; 54.5–100.0)	29 (29.5; 19.1–39.9)	21 (98.9; 96.6–100.0)	19 (81.1; 57.5–100.0)	17 (94.2; 82.7–100.0)
Spoke with peer educator or outreach worker, last 12 months								
Yes	149 (48.1; 41.5–54.8)	126 (86.4; 79.8–93.1)	103 (85.0; 78.2–91.8)	72 (74.4; 64.8–84.1)	36 (30.2; 20.3–40.1)	30 (93.3; 85.7–100.0)	24 (72.8; 51.9–93.7)	21 (86.1; 70.7–100.0)
No	177 (51.9; 45.2–58.5)	104 (74.3; 66.0–82.6)	84 (77.3; 67.0–87.6)	52 (64.0; 51.8–76.1)	71 (69.8; 59.9–79.7)	51 (90.2; 81.6–98.8)	44 (83.9; 71.1–96.7)	37 (88.6; 77.4–99.8)
Given free condoms, last 12 months								
Yes	232 (72.7; 67.0–78.3)	189 (86.3; 81.0–91.6)	154 (80.8; 74.0–87.6)	100 (69.5; 60.8–78.1)	59 (53.0; 42.2–63.9)	46 (87.4; 77.6–97.2)	36 (72.9; 56.2–89.5)	29 (77.4; 61.2–93.7)
No	95 (27.3; 21.7–33.0)	40 (59.7; 46.2–73.1)	33 (86.2; 75.5–96.9)	23 (71.4; 54.4–88.4)	49 (47.0; 36.1–57.8)	36 (96.6; 90.9–100.0)	33 (89.1; 76.4–100.0)	29 (95.5; 88.3–100.0)

Abbreviations: *CI* confidence interval

On multivariable analysis, in Juba, being aware of one’s HIV status was associated with being away from home for ≥1 month in the last 6 months (adjusted odds ratio [aOR], 2.7 [95% CI: 1.0–7.3]), not being ashamed of selling sex (aOR, 2.0 [95% CI: 1.0–3.9]), and receiving free condoms in the last 12 months (aOR, 3.0 [95% CI: 1.5–6.1]; Table 3). In Nimule, no variables were significant on multivariable analysis, but on bivariate analysis, awareness of HIV-positive status was higher among those who had at least a high school education than those with no education and among those who did not use a condom at last sex (Table 4).

**Table 3:** Correlates of being aware of HIV-positive status among female sex workers in Juba, South Sudan, 2016

	Juba			
Variable	OR (95% CI)	***p***-value	aOR (95% CI)	***p***-value
Nationality		0.0343		0.3102
Other	2.83 (1.03–7.78)		1.68 (0.61–4.62)	
South Sudanese	Ref		Ref	
Away from home for ≥1 month, past 6 months		0.0114		0.0449
Yes	3.06 (1.31–7.16)		2.73 (1.02–7.31)	
No	Ref		Ref	
Disclosed sex work to anyone^a^		0.0577		0.2141
No	2.07 (1.01–4.25)		1.63 (0.75–3.55)	
Yes	Ref		Ref	
Ashamed of selling sex		0.0218		0.0576
No	2.79 (1.29–6.05)		2.0 (1.0–3.9)	
Yes	Ref		Ref	
Used condoms with all male clients, last 6 months		0.0249		0.2637
Yes	2.18 (1.09–4.36)		1.51 (0.73–3.12)	
No	Ref		Ref	
Used condom at last sex act		0.0329		0.4556
Yes	2.87 (1.05–7.89)		1.46 (0.54–3.95)	
No	Ref		Ref	
Spoke with peer educator or outreach worker, last 12 months		0.0234		0.3827
Yes	2.20 (1.08–4.50)		1.39 (0.66–2.94)	
No	Ref		Ref	
Given free condoms, last 12 months		0.0001		0.0027
Yes	4.25 (2.08–8.71)		3.00 (1.47–6.14)	
No	Ref		Ref	

Abbreviations: *OR* odds ratio; *aOR* adjusted odds ratio; *CI* confidence interval

^a^ Includes immediate and extended family members, spouse or partner, friends who do not sell sex, health care providers, and other

**Table 4:** Variables significant in bivariate analysis by awareness of HIV-positive status among female sex workers in Nimule, South Sudan, 2016

	Nimule			
Variable	OR (95% CI)	***p***-value	aOR (95% CI)	***p***-value
Education		0.0280		0.1011
No formal education	0.12 (0.01–1.20)		6.38 (0.51–80.28)	
High school or higher	0.04 (0.00–0.40)		0.34 (0.05–2.50)	
Primary	Ref		Ref	
Away from home for ≥1 month, past 6 months		0.0856		0.4250
No	3.95 (0.82–18.98)		2.19 (0.31–15.31)	
Yes	Ref		Ref	
Used condom at last sex act		0.0328		0.5255
No	11.37 (1.23–105.53)		2.53 (0.14–25.42)	
Yes	Ref		Ref	

Abbreviations: *OR* odds ratio; *aOR* adjusted odds ratio; *CI* confidence interval

## Discussion

Although awareness of HIV-positive status, ART uptake, and VL suppression is higher among the general population than FSW in many countries, our findings reveal the opposite in South Sudan [10]. Additional services are still needed, however, to reach 90–90-90 targets among FSW in South Sudan. In Juba, attainment of the first two 90 targets for FSW and sexually exploited female adolescents by 2020 appears feasible, and the first 90 target has already been achieved in Nimule. Data from other countries also indicate a need for improved case finding to increase awareness of HIV-positive status because once HIV is diagnosed, most people are linked and retained on ART and consequently reach viral suppression [10]. Our findings, in contrast, reveal gaps in ART uptake and viral suppression among FSW and sexually exploited female adolescents, particularly in Juba.

The high proportion of non-South Sudanese FSW and sexually exploited female adolescents living with HIV may contribute to these findings because foreign FSW and sexually exploited female adolescents may have received services in their home countries. This may also explain why coverage was higher in Nimule than Juba even in the immediate post-conflict period during which HIV services in Nimule were interrupted. Inconsistent ART use could be due in part to an inability to return to their home country to obtain more medicine and may contribute to lower HIV service utilization and low viral suppression. This may further explain the differences in coverage between Juba and Nimule. Although 66.7% of FSW and sexually exploited female adolescents in Juba were from Uganda compared to 36.8% in Nimule, those in the border city Nimule can more easily return to Uganda for services [14, 15]. It is even harder for Congolese and Kenyans, who account for 17.0 and 10.0%, respectively, of FSW and sexually exploited female adolescents living with HIV in Juba, to return to their home countries to obtain ART because of the distance. Less than 2% of FSW and sexually exploited female adolescents living with HIV in Nimule were Congolese or Kenyan. Additional support to retain FSW and sexually exploited female adolescents on ART may be warranted in South Sudan.

The different correlates of awareness of HIV-positive status that we identified may result from the different contexts and FSW and sexually exploited female adolescents populations in Juba and Nimule, which underscores the importance of conducting surveys in multiple locations and tailoring services accordingly. For instance, in Juba, awareness of HIV status was higher among FSW and sexually exploited female adolescents who had been away from home for ≥1 month in the last month. This may be because women feel more comfortable testing for HIV when away from home.

Targets of stigma may internalize feelings of shame [25]. Our finding that FSW and sexually exploited female adolescents in Juba who were not ashamed of selling sex were more likely to be aware of their HIV-positive status are consistent with findings that stigma and shame impede HIV service utilization and are associated with high HIV prevalence [6, 26–28]. FSW and sexually exploited female adolescents who were ashamed of selling sex may have experienced more stigma than those who were not ashamed. The illegal nature of sex work in South Sudan may have a dual role in inhibiting healthcare access by FSW and sexually exploited female adolescents by rendering them afraid of arrest and exacerbating stigma.

Though FSW and sexually exploited female adolescents in Juba who were aware of their status were more likely to be given free condoms, they were not more likely to use condoms with clients or at last sex. Given the timing of our survey and the low level of comprehensive HIV knowledge, it is unlikely that FSW and sexually exploited female adolescents were aware that suppressed viral load can prevent HIV transmission and consequently chose not to use condoms. It is more likely that, after receiving an HIV-positive diagnosis and initiating ART, FSW and sexually exploited female adolescents were given condoms by healthcare providers. This suggests that FSW and sexually exploited female adolescents may not be receiving condoms from peer educators. Additionally, because awareness of HIV status did not differ between FSW and sexually exploited female adolescents who engaged with a peer outreach worker in the last 12 months and those who had not, substantial gains in service provision could be achieved by improving the quality of interactions with peer educators and outreach workers and expanding these programs to include the provision of condoms and HIV testing or linkage to testing. Peer educators and outreach workers can further support FSW and sexually exploited female adolescents who have already received an HIV-positive diagnosis to ensure they are linked and retained on treatment.

The low proportion of FSW and sexually exploited female adolescents in Juba who are on ART and have suppressed viral load is of concern. This may be because FSW programs do not provide ART but only provide referral to ART sites where FSW and sexually exploited female adolescents may not receive the differentiated services they need because they fear stigma. An additional factor may be HIV drug resistance. In the absence of drug resistance testing, service providers could solicit information about barriers to ART adherence from FSW and sexually exploited female adolescents provide differentiated services including convenient ART dispensing options, enhanced adherence counseling, and transfer to fixed-dose tenofovir/lamivudine/dolutegravir.

Our survey is limited by the cross-sectional nature of our data, and our relatively small sample sizes limit our ability to assess associations with each of the 90–90-90 targets. Given the multinational character of FSW and sexually exploited female adolescents in Juba and Nimule, future surveys could ask participants in which country they obtain HIV testing and treatment services and reasons for obtaining services outside of South Sudan. We were unable to recruit Ethiopian and Eritrean FSW and sexually exploited female adolescents in both surveys. There may also be response bias due to the face-to-face nature of our interviews. Our sensitivity analysis of the 90–90-90 results sought to address this for these three variables.

## Conclusions

Though progress toward 90–90-90 targets is greater among FSW and sexually exploited female adolescents than among the general population in South Sudan, this may be because of the perceived high risk of HIV acquisition among FSW and sexually exploited female adolescents, which may encourage FSW and sexually exploited female adolescents to seek HIV services in contrast to the general population. Limited access to HIV services in the country may also contribute to the low service coverage in the general population, whereas FSW and sexually exploited female adolescents lived in towns with better access to HIV services. Targeting South Sudan’s limited resources based on HIV burden by location and populations could increase the impact of these HIV programs and help control the spread of HIV. The high awareness of HIV status among FSW and sexually exploited female adolescents suggest that testing programs that target these women and girls are effective and can have a substantial impact if expanded to reach all FSW and sexually exploited female adolescents.

## Data Availability

To further protect survey participants the dataset supporting the conclusions of this article is available by reasonable request to the corresponding author.
